# Influence of Rotational Speed on Accuracy in Measuring MIRS of Aerospace Aluminum Alloy by Incremental Hole-Milling Method

**DOI:** 10.3390/ma18071582

**Published:** 2025-03-31

**Authors:** Junbo Shen, Hailin He, Chen Li, Youping Yi, Shiquan Huang

**Affiliations:** 1 College of Mechanical and Electrical Engineering, Central South University, Changsha 410083, China; 2 Research Institute of Light Alloy, Central South University, Changsha 410083, China; 3 Xi’an Aircraft Design and Research Institute, Xi’an 710000, China

**Keywords:** machining-induced residual stress, incremental hole-milling method, rotational speed, shear stress, distortion

## Abstract

The incremental hole-drilling method is widely used to measure residual stress distribution versus depth. In this study, a two-flute carbide end mill that was 1.5 mm in diameter operated at different rotational speeds, used to create a hole that was 2.0 mm in diameter through orbital technology milling, were used to measure machining-induced residual stress (MIRS). Additionally, a finite element model was developed to calculate distortion, with MIRS as the input. A 25 × 25 × 1 mm^3^ thin sample containing a machining surface was cut free from large samples by a wire electrical discharge machining after milling, and distortion was measured by a 3D profile meter. It can be concluded that the calculated maximum distortion on one of the diagonals can reach 89% of the measured maximum distortion when the rotational speed is more than 20,000 rpm, and the deviation in the measured MIRS can be controlled within 35 MPa. The shear stress increases rapidly by 63% when the rotational speed is less than 10,000 rpm.

## 1. Introduction

MIRS on distorted parts cannot be ignored in aerospace thin-walled aluminum components [1,2,3], constituting a process related to the formulation of subsequent machining process routes and that ultimately affects the dimensional accuracy and stability of the final workpieces [4]. However, there are still some difficulties in measuring MIRS because it is only located a few μm beneath the machining surface, and the actual MIRS usually has a non-uniform distribution at different depths [5].

At present, common methods such as incremental hole drilling [6], X-ray diffraction combined with electrolytic polishing [7] and slotting [8] are used to measure stress distribution versus depth in aluminum alloys. However, electrolytic polishing cannot control the depth of the measured parts precisely; thus, it is very difficult to assess the actual stress distribution when it relates to spatial gradient stress. Slotting methods produce a long slot in the machining surface, which results in a large amount of damage to the parts. Incremental hole-drilling methods only produce a hole of about 2.00 mm, resulting in little damage to the workpieces; because of this huge advantage, they have become the most widely used techniques in characterizing uniform and non-uniform stress in materials beyond aluminum.

Despite the huge advantage of the incremental hole-drilling method, its repeatability and the accuracy of its measurement results are influenced by many factors, such as calibration coefficients, rotational speed, tools, the deviation of the actual geometry from the ideal size of a hole, the accuracy of each incremental step, the operator’s experience and so on. Chighizola [8] used the hole-drilling method, X-ray diffraction combined with the electrolytic polishing and the slotting method to measure MIRS in aluminum. The results showed that the accuracy and repeatability of the measurement results obtained by the hole-drilling and slotting methods were better than the X-ray diffraction method when the depth of the milled surface was greater than 0.05 mm. On the contrary, the accuracy and repeatability of the measurement results obtained by the X-ray diffraction method for a shallow surface between 0 and 0.050 mm was better than the hole-drilling and slotting methods. D. Peral [9] developed a novel method to correct the eccentricity of holes, which improved the accuracy in the order of a few tenths of a percent. Nau’s studies [10] showed that the real stress state was not influenced when the air pressure to power the air turbine was set to 3 bar and 4 bar (rotational speed of 300,000 rpm and 400,000 rpm, respectively) by using orbital technology. Steinzig’s studies [11] showed that high accuracy and good repeatability measurement results can be obtained when the rotational speed is between 10,000 and 40,000 rpm at a feed rate of 0.05 mm/s by using two-tooth end mills for an aluminum alloy and stainless steel. Schajer [12] developed a numerical method to acquire calibration data in accordance with the length and width of various strain rosettes, resulting in a ~2% deviation in the actual value of the custom computation results based on the strain rosette geometry, which eventually caused a ~3% deviation in computational stress.

Although research on stress measurement by the incremental hole-drilling method is relatively developed, an ultra-high speed between 300,000 and 400,000 rpm is mainly used; there is rarely a systematic focus on rotational speeds less than 30,000 rpm. There is also a lack of experimental verification considering the coupling effects of normal stress and shear stress. In this work, measuring the distribution versus depth of MIRS in milled aluminum under different rotational speeds, especially under 30,000 rpm, is conducted by incremental hole-milling methods using orbital milling technology to figure out to what extent rotational speed influences the measured stress. At the same time, a finite element model considering both measured normal and shear stress as input is also developed to calculate distortion. Experimental verification is then conducted after finishing the measurement of MIRS, where a sample cube of 25 × 25 × 1 mm^3^ containing a machining surface is removed from large samples by wire electrical discharge machining; then, the cube sample is scanned by a 3D profile meter to acquire the actual machining distortion, which is considered to have only been caused by MIRS. The measured and calculated distortions are compared to verify the accuracy and repeatability of the measured stress. Two finite element models only considering normal stress and shear stress separately are also developed to elucidate the influence of normal and shear stress on distortion.

## 2. Materials and Methods

### 2.1. Sample Preparation

An aerospace aluminum alloy named 7XXX-T7451 manufactured by SOUTHWEST ALUMINUM (GROUP) Co., Ltd. which is located in Chongqing, China with a thickness of 80 mm was selected to conduct experiments. Samples with a size of 240 × 80 × 35 mm^3^ were cut by sawing from plates with a size of 2000 × 1500 × 80 mm^3^, where the 240 mm dimension indicates the longitudinal direction, the 80 mm dimension indicates the thickness direction and the 35 mm dimension indicates the transverse direction. Experiments were carried out on the surface of 240 × 80 mm^2^. The local coordinate system is defined on the samples, where the 240 mm dimension represents the x direction, the 80 mm dimension represents the y direction and the 35 mm dimension represents the z direction.

### 2.2. Machining Experiment

Samples were clamped by a vice, length direction of the samples was parallel to the jaw, and the width of clamping area was about 120 mm. Machining experiments were carried out with a thickness of 5 mm above the jaw along the z direction. The length direction of the samples was parallel to the x direction of the machining tool coordinate system, the width direction of the samples was parallel to the y direction of the machining tool coordinate system, and the height direction of the samples was parallel to the z direction of the machining tool coordinate system. Aerospace-grade three-tooth carbide end mills, 12 mm in diameter, whose spiral angle and fillet radius were 45° and 0 mm, respectively, were used. Machining experiments were carried out on HERMLE C30U five-axis machining center manufactured by HERMLE which is located in Gosheim, German. The Vc representing cutting speed was 200 m/min. The depth of cut a_p_ and the width of cut a_e_ were set to 3 mm and 4 mm, respectively. The feed per tooth of f_z_ was held constant at 0.20 mm. Down milling and a single feed direction were chosen. Tools and parts were cooled sufficiently to prevent tool wear and surface performance changes in the samples caused by temperature deviation.

### 2.3. Residual Stress Measurement

The incremental hole-milling method was chosen to conduct MIRS measurements by strain gauge methods according to the latest standard ASTM E837-20 issued by the American Society of Materials. This type of strain rosette contains three sets of grids, and the angles between adjacent grids are 0°, 90° and 225°, respectively. The mean diameter of the strain rosette is 5 mm. The strain rosette was pasted on the surface of parts and connected to the static strain gauge. As material is removed layer by layer, strains along the three directions of 0°, 90° and 225° are recorded. According to functional relationship between stress and strain versus depth derived from ASTM E837-20 [13], MIRS distribution versus depth can be calculated. According to research by many scholars [14,15], MIRS changes sharply in the shallow surface at 0~0.20 mm and gradually slows down with the increase in depth. Therefore, a fine incremental step was adopted at the shallow surface, and as the depth increased, the incremental step gradually became larger and larger. Specifically, 10 incremental steps were set to 0.013 mm, 6 incremental steps were set to 0.026 mm, and 6 incremental steps were set to 0.052 mm; thus, a final depth of 0.598 mm in total was reached. At the same time, orbital milling technology based on a high-accuracy CNC machining center was adopted, and the axial feed speed of F was set to 0.001 mm/s in order to reduce additional MIRS. Thus, a hole which was 2.0 mm in diameter and a final depth of 0.598 mm were achieved by using a two-flute carbide end mill which was 1.5 mm in diameter through orbital milling technology. The positioning accuracy of the measurement instrument is 1 μ, which fully meets the required machining parameters.

**Figure 1:**
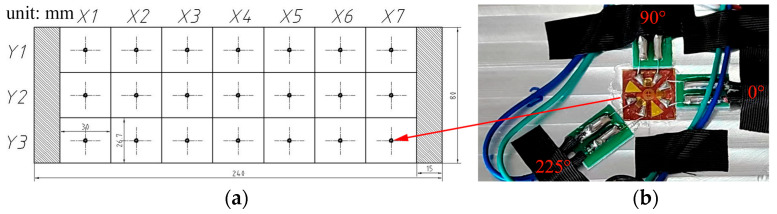
Measurements of MIRS through incremental hole-milling method: (**a**) Samples mesh layout, where X#Y# indicates the location of the grid. A total of 15 mm on both sides of the sample are excluded. unit: mm. (**b**) Strain gauge rosette arrangement for conducting incremental hole-milling method near the center of each mesh.

Figure 1 shows the locations of the measuring point of MIRS. The milled surface of the samples was divided into 7 grid units and 3 grid units along the longitudinal and transverse direction, respectively, taking into account the convenience of pasting strain rosettes and the connection of the strain rosettes and the statical strain indicator. The MIRS measurement points were located near the center of each grid. About 15 mm parallel to feed direction on both sides was removed to exclude an unstable situation of MIRS.

The rotational speed has an important influence on the accuracy and repeatability of MIRS, so different rotational speeds were set in order to measure the distribution of MIRS. Table 1 shows experimental parameters such as rotational speed, tool diameter, hole diameter and each measurement point location.

### 2.4. Experimental Verification

**Figure 2:**
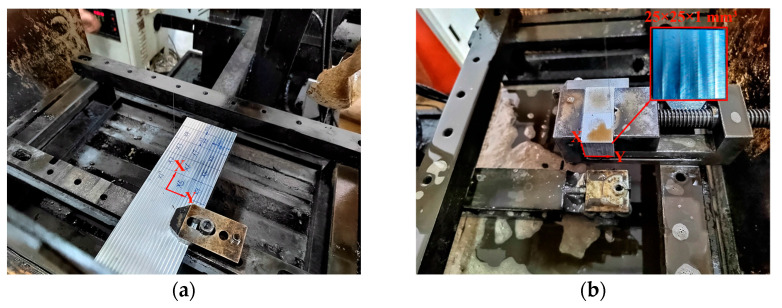
Wire electrical discharge machining process: (**a**) removal of 25 mm^3^ cube from large parts and (**b**) removal of 25 × 25 × 1 mm^3^ parts from the 25 mm^3^ cube.

The accuracy and repeatability of MIRS were evaluated by conducting machining experiments. Figure 2 shows the processes of the samples cut by wire electrical discharge machining (EDM). Firstly, a cube of 25 mm^3^ was removed from the large, milled samples whose size were 240 × 80 × 32 mm^3^ by EDM. Secondly, the cube was rotated by 90°; then, a sample whose size was 25 × 25 × 1 mm^3^ containing the milling surface was obtained. Then, the sample was scanned at an interval of 0.02 mm along the longitudinal and transverse directions by a 3D profile meter, and a distortion color map out of the plane was obtained. The distortion could be considered to be caused completely by MIRS.

**Figure 3:**
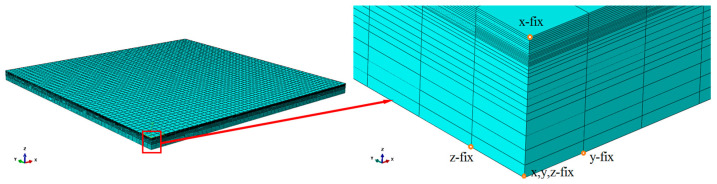
Finite element model of sample and its boundary conditions.

Figure 3 shows the finite element model and its boundary based on actual samples, whose size is 25 × 25 × 1 mm^3^. MIRS changes dramatically in the depth layer of 0~0.2 mm beneath a milled surface according to the literature [16], so the mesh near the surface was refined to 0.01 mm and became coarser and coarser with the increase in depth. The approximate global size of the elements was 0.5 mm. An 8-node linear brick element, C3D8R, was used for meshing by the structured technique, and there was a total of 80,000 elements. A linear elastic constitutive model with an elastic modulus of 71,590 MPa and a Poisson’s ratio of 0.33 was used to calculate machining-induced distortion. The x direction, the y direction, the z direction and the x, y, z directions of four different nodes in turn which are not in the same plane were constrained, which allowed the model to deform freely and could also prevent the model from rigid displacement, which causes difficulty in calculation convergence. MIRS versus depth was applied into each centroid of the element grid by linear difference methods. The distortion data out of the z direction of the finite element model were extracted after completing calculation. The accuracy and repeatability of MIRS were evaluated by comparing the measured and calculated distortion data.

## 3. Results

### 3.1. The Influence of Rotational Speed on Residual Stress Measurements

**Figure 4:**
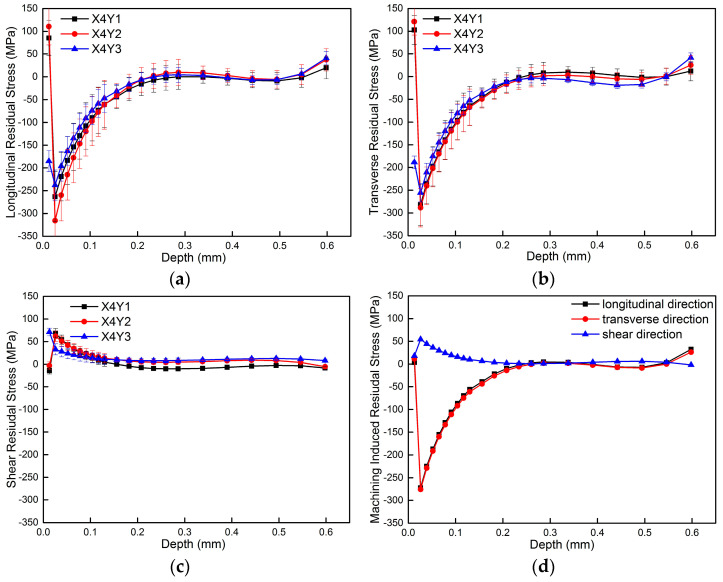
Measurement results of MIRS at the rotational speed of n = 30,000 rpm. (**a**) Longitudinal residual stress of X4Y1, X4Y2 and X4Y3. (**b**) Transverse residual stress of X4Y1, X4Y2 and X4Y3. (**c**) Shear residual stress of X4Y1, X4Y2 and X4Y3. (**d**) Average MIRS of X4Y1, X4Y2 and X4Y3.

Figure 4 shows results of MIRS versus depth when a hole which is 2 mm in diameter is obtained through orbital milling technology using a two-flute carbide end mill of 1.5 mm in diameter at the rotational speed of 30,000 rpm. It can be seen that MIRS values versus depth along the longitudinal and transverse directions are compressive and show a √ type distribution. The maximum stress could be up to −300 MPa at the location of −0.026 mm beneath the milled surface. The shear stress is tensile and shows a type of parabola distribution. The maximum shear stress is about 60 MPa at the location of −0.026 mm beneath the milled surface. The standard deviation in MIRS could be controlled within 35 MPa at the depth of 0~−0.130 mm beneath the milled surface. The standard deviation in MIRS could be controlled within 15 MPa when the depth was more than −0.13 mm beneath the milled surface, which indicates that high-accuracy measurement results could be obtained through the incremental hole-milling method. In addition, it can be seen clearly from Figure 4 that depth of MIRS is about 0.2 mm. The stress level is close to the initial bulk residual stress (IBRS) when it is greater than 0.2 mm. Finally, the MIRS values measured at three different locations are in good agreement, which indicates that the incremental hole-milling method has good repeatability.

**Figure 5:**
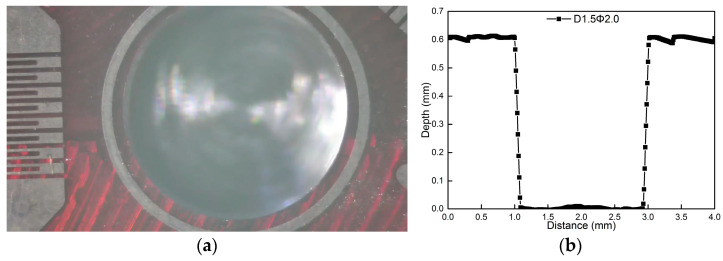
Measurement results at rotational speed of 30,000 rpm for a hole whose size is 2.0 mm in diameter: (**a**) macrostructure of hole (**b**) hole profile versus depth.

Figure 5 shows the macrostructure and profile versus depth of a hole of size 2.0 mm in diameter formed at the rotational speed of 30,000 rpm. It is evident form Figure 5 that a regular hole perimeter is obtained. The diameter and depth of the measured hole by a laser profile instrument are 2.000 mm and 0.608 mm, respectively, and the standard deviations are 0.000 mm and 0.010 mm, respectively. A regular hole shape and precise hole depth are essential factors to achieve high accuracy and repeatability measurement results, as shown in Figure 5, by the incremental hole-milling method.

**Figure 6:**
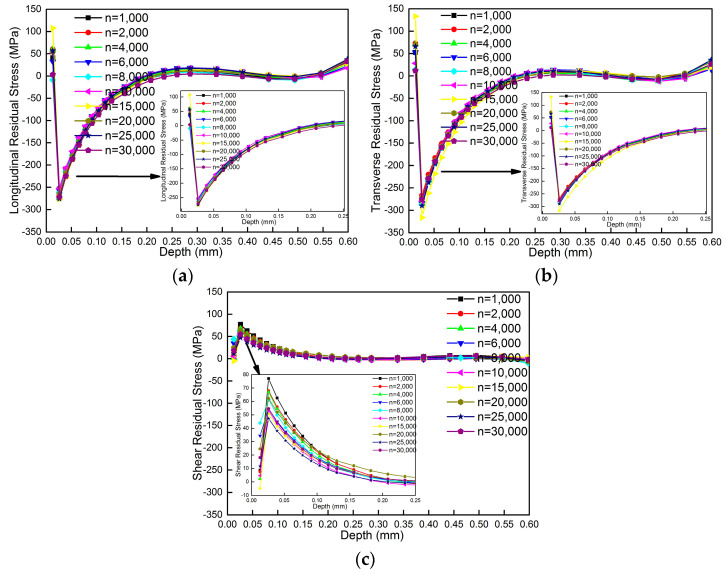
Measurement results of MIRS obtained under different rotational speeds. (**a**) Longitudinal residual stress. (**b**) Transverse residual stress. (**c**) Shear residual stress.

Figure 6 shows the average measurement results of MIRS at three different locations obtained at rotational speeds of 1000 rpm, 2000 rpm, 4000 rpm, 6000 rpm, 8000 rpm, 10,000 rpm, 15,000 rpm, 20,000 rpm, 25,000 rpm and 30,000 rpm. It can be seen from Figure 6 that distribution of MIRS achieved under different rotational speeds is consistent. The longitudinal and transverse residual stress values versus depth are compressive and show a √ type of distribution. The shear residual stress is tensile, showing a parabola-type distribution. The depth of residual stress is about 0.2 mm. The influence of rotational speed on MIRS along the longitudinal and transverse directions is not significant, which mainly affects the distribution of shear residual stress. As rotational speed decreases, shear residual stress gradually increases. The maximum shear residual stress is 77.07 MPa when the rotational speed is 1000 rpm. The shear residual stress increases as high as 63% when it is compared with measurement results at the rotational speed of 25,000 rpm. The reason is that with the decrease in rotational speed, the extra stress introduced by the hole-milling method increases.

### 3.2. The Influence of Rotational Speed on Machining Distortion Results

**Figure 7:**
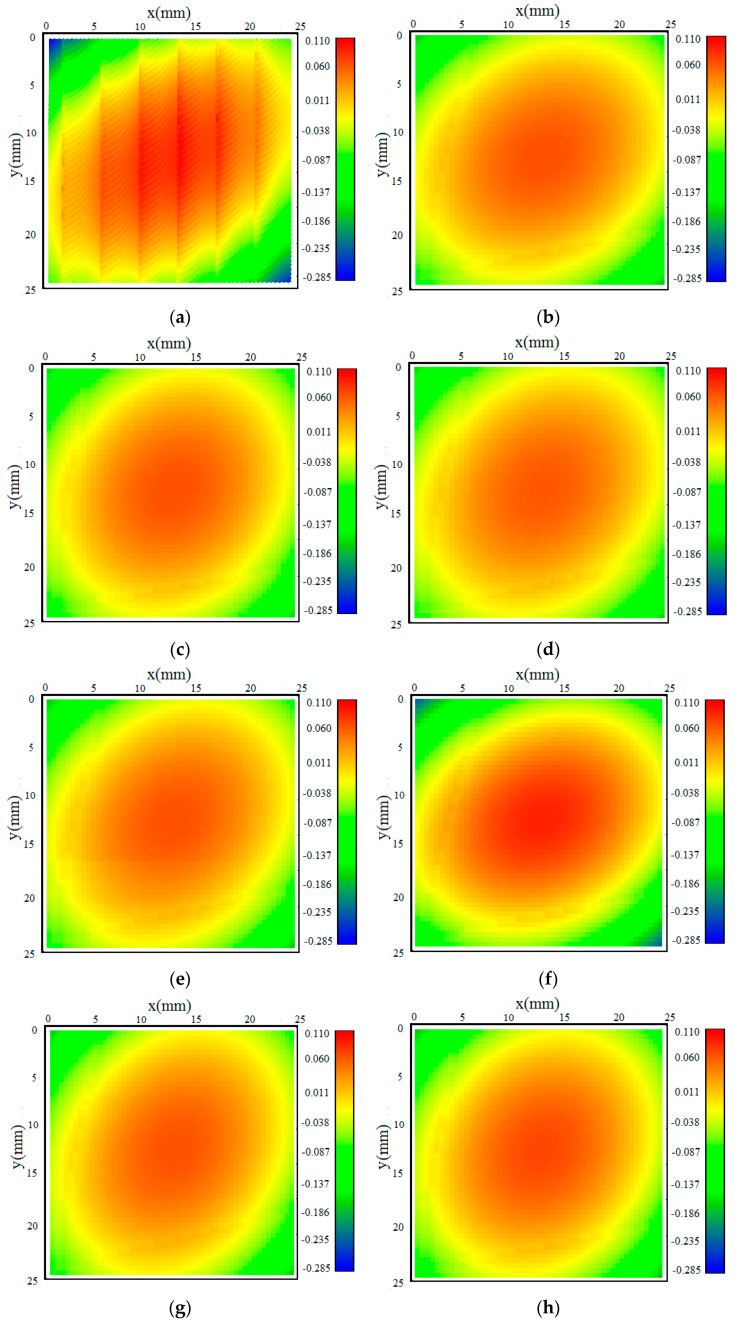

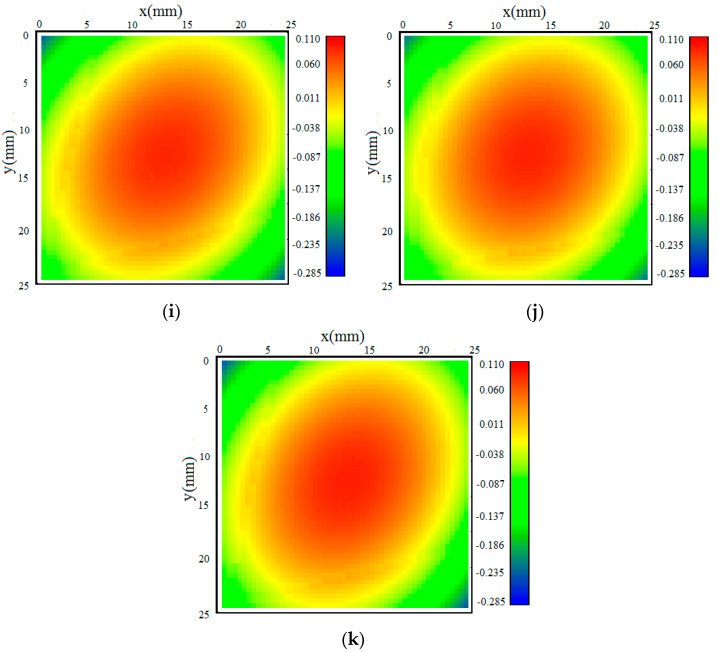
Color maps: (**a**) measured distortion; (**b**) n = 1000 rpm; (**c**) n = 2000 rpm; (**d**) n = 4000 rpm; (**e**) n = 6000 rpm; (**f**) n = 8000 rpm; (**g**) n = 10,000 rpm; (**h**) n = 15,000 rpm; (**i**) n = 20,000 rpm; (**j**) n = 25,000 rpm; (**k**) n = 30,000 rpm.

Figure 7 shows color maps of measured and calculated results with measured stress as input at the rotational speeds of 1000 rpm, 2000 rpm, 4000 rpm, 6000 rpm, 8000 rpm, 10,000 rpm, 15,000 rpm, 20,000 rpm and 30,000 rpm. It can be seen from Figure 7 that the measured color map represents the milled surface, with convex distortion in the middle and concave distortion at the corners, in the z direction when a thin sample is cut free from large samples. The largest distortion occurs along the line from (0, 0) to (25, 25) mm. The color maps of the calculated distortion with the calculated stress as input have a closer similarity to the measured distortion when the rotational speed is more than 20,000 rpm.

**Figure 8:**
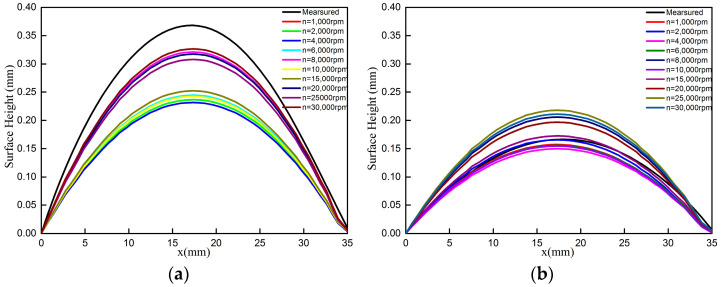
Line plots of measured and calculated color maps: (**a**) (0, 0) to (25, 25) mm and (**b**) (0, 25) to (25, 0) mm.

Figure 8 shows line plots of the measured and calculated distortion from (0, 0) to (25, 25) mm and from (0, 25) to (25, 0) mm, respectively. The shapes of the measured and calculated displacement curves agree well in general. In the line plots from (0, 0) to (25, 25) (Figure 8a), the measured distortion is a peak-to-valley distortion of 0.368 mm, while the calculated results under different rotational speeds show peak-to-valley distortions of 0.244 mm, 0.237 mm, 0.232 mm, 0.244 mm, 0.321 mm, 0.242 mm, 0.252 mm, 0.317 mm, 0.308 mm and 0.327 mm, and the deviations between the measured distortions and the calculated values are 0.124 mm, 0.131 mm, 0.136 mm, 0.124 mm, 0.047 mm, 0.126 mm, 0.116 mm, 0.051 mm, 0.060 mm and 0.041 mm, respectively. Along the line from (0, 25) to (25, 0) (Figure 8a), the measured distortion is a peak-to-valley distortion of 0.166 mm, while the calculated results under different rotational speeds show peak-to-valley distortions of 0.157 mm, 0.166 mm, 0.150 mm, 0.156 mm, 0.206 mm, 0.154 mm, 0.173 mm, 0.197 mm, 0.218 mm and 0.211 mm, and the deviations between the measured distortions and the calculated values are 0.009 mm, 0.000 mm, 0.016 mm, 0.010 mm, 0.040 mm, 0.012 mm, 0.007 mm, 0.031 mm, 0.052 mm and 0.045 mm, respectively. The maximum displacement value of the measured and calculated data along the line from (0, 0) to (25, 25) mm is more than twice that along the line from (0, 25) to (25, 0) mm, which indicates that the samples exhibit torsional distortion. Based on the measured and calculated distortion results, it can also be seen that the largest relative error is less than 0.060 mm when a rotational speed of more than 20,000 rpm is used in the process of MIRS measurement. The maximum distortion on one of diagonals of the calculated results accounts for 86%, 84% and 89%, compared with the maximum diagonal distortion of the measured data, which also indicates that the measured stress is almost equal to the actual distribution of the MIRS.

## 4. Discussion

**Figure 9:**
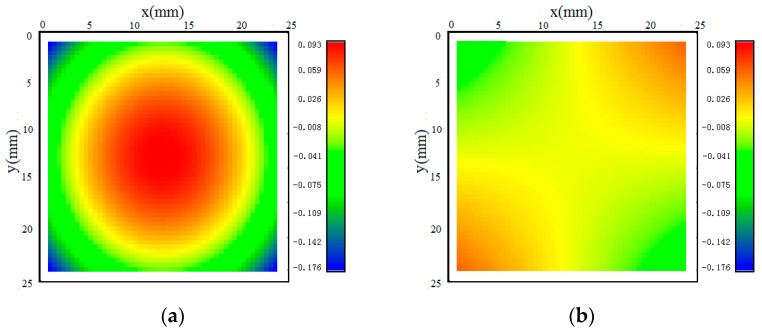
Color maps of calculated distortion when n = 30,000 rpm: (**a**) only considering normal stress σ_xx_ and σ_yy_ and (**b**) only considering shear stress τ_xy_.

**Figure 10:**
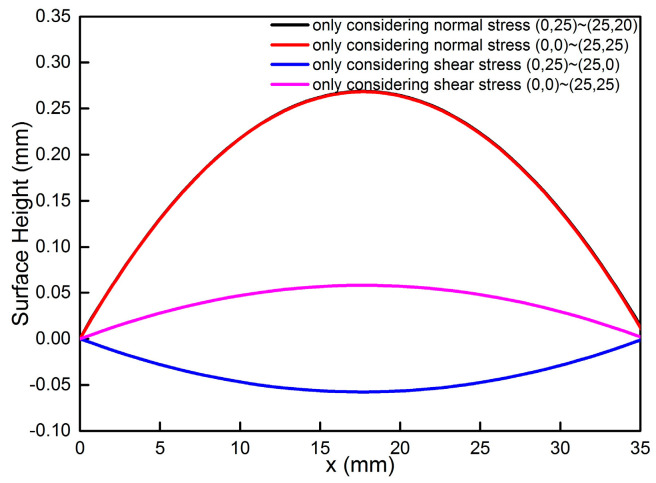
Calculated distortion from corner to corner considering normal stress and shear stress separately when n = 30,000 rpm.

The work reported here shows the influence of rotational speed on the measurement results of MIRS versus depth in aluminum parts milled by incremental hole-milling technology. There is a significant increase in shear residual stress when the rotational speed decreases, especially when the rotational speed is below 10,000 rpm. We aimed to figure out to what extent shear stress contributes to distortion caused by MIRS. Figure 9 shows the calculated distortion only considering normal stress, which is represented by σ_xx_ and σ_yy_, and shear stress, i.e., τ_xy_, which has been rarely reported in the literature before. It can be clearly seen that the peak distortion in Figure 10 is 0.269 mm on both diagonals when considering only normal stress, and it is bowl-shaped. The maximum distortions in Figure 10 are −0.058 mm and 0.058 mm along the lines from (0, 25) to (25, 0) mm and from (0, 0) to (25, 25) mm, respectively, and they show a saddle shape. The combined interaction between normal stress and shear stress generates the elliptical shape rotating away from the feed direction, as shown in Figure 7. Thus, it can be concluded that shear stress plays an important role in producing the actual distortion shape of the samples.

**Figure 11:**
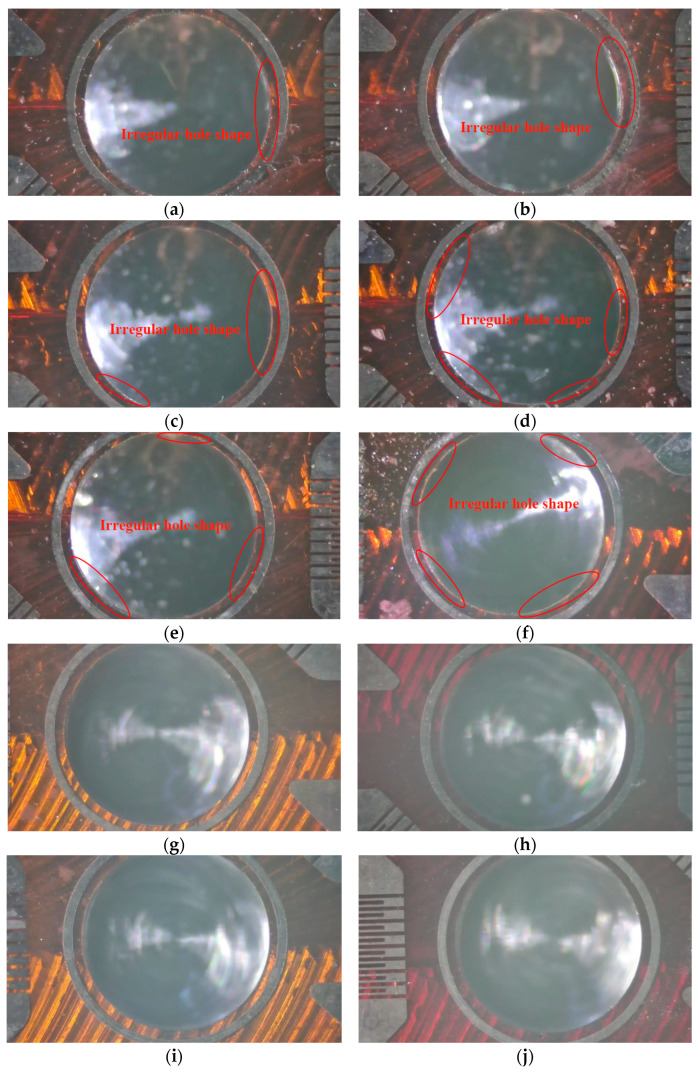
Images of a hole of 2.0 mm in diameter under rotational speeds of (**a**) 1000 rpm, (**b**) 2000 rpm, (**c**) 4000 rpm, (**d**) 6000 rpm, (**e**) 8000 rpm, (**f**) 10,000 rpm, (**g**) 15,000 rpm, (**h**) 20,000 rpm, (**i**) 25,000 rpm and (**j**) 30,000 rpm.

**Figure 12:**
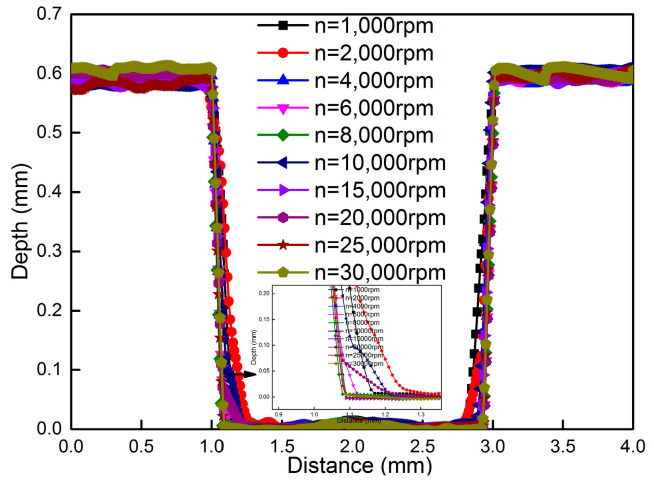
Hole depth profile under different rotational speeds.

The reason may be that as rotational speed decreases, the hole-making process could induce more residual stress. It can also be seen from Figure 11 that uniformity of the hole shape is observed when a rotational speed of more than 15,000 rpm is used. The quality of the hole perimeter becomes worse at less than 15,000 rpm and becomes quantitatively even worse when RPM is reduced further. The degradation of the hole macrostructure results in more and more standard deviation in the measurement results, which is consistent with the result showed in Figure 6, which indicates that shear residual stress increases by 63% at the rotational speed of 1000 rpm. Figure 12 shows the hole depth profile scanned by the laser profile meter under different rotational speeds. It can be seen that measured depth ranges from 0.591 to 0.608 mm and the maximum standard deviation in hole depth is only 0.010 mm compared with the ideal value of 0.598 mm, which indicates that hole depth is not apparently influenced by rotational speed. But it can be clearly seen from Figure 12 that a larger round radius deviation appears when the rotational speed is less than 15,000 rpm, and the deviation compared with ideal value of 2.000 mm is in the range 0.19~0.35 mm at the bottom. Finally, shear residual stress and deviation in hole shape apparently increase at rotational speeds of less than 15,000 rpm, which is mainly because, as rotational speed decreases, the interaction force between end mills and materials increases, resulting in a non-uniform hole.

## 5. Conclusions

The MIRS of an aerospace aluminum alloy after milling can be obtained accurately by the incremental hole-milling method, which provides a basis for controlling distortion and optimizing the process. The following conclusions can be drawn:

(1) Rotational speed has an important influence on the accuracy and repeatability of MIRS. When the rotational speed reaches more than 20,000 rpm, the deviation in MIRS can be controlled within 35 MPa. The rotational speed mainly affects the shear stress. The shear stress increases significantly, and the maximum value increases by 63% when the rotational speed is less than 10,000 rpm.

(2) Considering the coupling effects of normal stress and shear stress, distortion experiments and finite element simulation using measured stress as input are conducted, respectively. The results show that the maximum calculated distortion on one of the diagonal lines can reach 89% of the measured diagonal line values when the rotational speed is more than 20,000 rpm.

## Figures and Tables

**Table 1:** Summary of parameters used to measure MIRS of aluminum alloy 7XXX-T7451 including rotational speed, tool diameter, hole diameter and measurement point locations.

Serial Number	Rotational Speed, rpm	Tool Diameter, mm	Hole Diameter, mm	Measurement Point Location
1	1000	1.5	2.0	X1Y1,X1Y2,X1Y3
2	2000	1.5	2.0	X2Y1,X2Y2,X2Y3
3	4000	1.5	2.0	X3Y1,X3Y2,X3Y3
4	6000	1.5	2.0	X4Y1,X4Y2,X4Y3
5	8000	1.5	2.0	X5Y1,X5Y2,X5Y3
6	10,000	1.5	2.0	X6Y1,X6Y2,X6Y3
7	15,000	1.5	2.0	X1Y1,X1Y2,X1Y3
8	20,000	1.5	2.0	X2Y1,X2Y2,X2Y3
9	25,000	1.5	2.0	X3Y1,X3Y2,X3Y3
10	30,000	1.5	2.0	X4Y1,X4Y2,X4Y3

## Data Availability

The original contributions presented in this study are included in the article. Further inquiries can be directed to the corresponding authors.
